# Reduced bacterial contamination rates detected on silicone tourniquets compared to conventional tourniquets in clinical routine

**DOI:** 10.1186/s12879-020-04975-y

**Published:** 2020-03-26

**Authors:** Marcus Grohmann, Lena Schomakers, Frank Wolschendorf, Janina Grosch, Susan Lindner, Anna Kristina Witte

**Affiliations:** HTK Hygiene Technologie Kompetenzzentrum GmbH, Heinrichsstr. 6, 96047 Bamberg, Germany

**Keywords:** Silicone tourniquets, Peripheral venous access, Blood sampling, HAI (hospital acquired infection), Infection control, Contamination

## Abstract

**Background:**

Tourniquets used for peripheral venous vascular access such as blood sampling are regularly contaminated in clinical routine. Although most contaminations are harmless, some pose a possible risk for infection. To improve peripheral venous access infection control standards, tourniquets with no or as few as possible bacterial burden should be used. Conventional tourniquets can be reprocessed by autoclaving or by incubating in disinfectants. However, both methods are time-consuming and not suitable for immediate use between patients. In contrast, silicone tourniquets can be quickly and simply reprocessed with wipe disinfection. In vitro studies from the manufacturer have demonstrated reduced bacterial contamination on silicone tourniquets after usage compared to conventional tourniquets. This study aims to independently investigate the bacterial load on both types of tourniquets in clinical routine.

**Methods:**

In a first trial, new conventional and silicon tourniquets were used for blood sampling in one facility with strict guidelines for reprocessing (after each patient or not at all) for 1 day and tested for bacterial contamination. In a second trial, new tourniquets were used in four facilities while the mode and frequency of tourniquets’ reprocessing was defined individually by each facility. The number of treated patients, mode and frequency of reprocessing and other relevant handling measures were documented.

**Results:**

Under controlled conditions, with strictly specified reprocessing, slightly fewer bacteria were found on silicone than on conventional tourniquets. In routine clinical practice the reprocessing frequency was not higher for silicone tourniquets in practice. Yet, in all four facilities, there were significantly fewer bacteria found on silicone than on conventional tourniquets.

**Conclusion:**

Although tourniquets are classified as non-critical medical devices, results show – together with benefits of faster and easier reprocessing – that silicone tourniquets can improve infection control of venous vascular access.

## Background

Blood sampling via peripheral venous access with the help of tourniquets is one of the most common invasive procedures in hospitals and other medical facilities. Due to contact with human skin and proximity to the puncture site, blood residues and bacterial contaminations were regularly found on tourniquets [1–3]. Besides environmental and skin microorganisms [4–6], the contamination can also include methicillin-resistant *Staphylococcus aureus* (MRSA) [1, 3, 7–12] or other multi-drug resistant bacteria [4, 13]. Hence, tourniquets may be a potential source for cross-contamination with potentially harmful pathogens. As hospital acquired infections (HAIs) cause substantial costs, increase length of hospital stay and mortality [14], strategies to prevent transmission of infectious agents should be applied.

There are two main strategies to address cross-contamination via tourniquets. First, several authors suggest to use disposable tourniquets [2, 3, 6]. But, sustainability, higher costs, handling issues and limitations in patient’s comfort are among the reasons why those are not regularly used [15]. The second strategy is based on reprocessing reusable tourniquets. They should be reprocessed after each patient, typically by autoclaving or incubation in disinfectants. However, both procedures are time consuming and thus, in practice reusable tourniquets are rarely processed between patients [3, 6, 16].

To increase tourniquet infection control compliance, innovative reusable tourniquets made out of silicone were developed (Suppl. Figure S1). According to the manufacturer’s instructions, disinfection of silicone tourniquets is only requiring wipe disinfection. In comparison to autoclaving or bathing in disinfectants, wipe disinfection is straightforward, fast and can be performed nearly anywhere. Based on the manufacturer’s information, there is minor bacterial contamination in comparison to conventional tourniquets after usage. To investigate whether this claim also holds true in clinical routine practice, we compared the contamination rate of used conventional to silicone tourniquets. Our results indicate that silicone tourniquets provide an opportunity to improve overall infection control and thereby patient safety in venous access procedures.

## Methods

### Study design

#### First trial

One outpatient clinic used alternately a conventional (CT) or silicone tourniquet (ST) for 1 day (five times). To receive an impression of bacterial contamination under controlled conditions guidelines were given for cleaning tourniquets with disinfectant wipes - either never (1) or each time (2) after blood sampling.

#### Second trial

To obtain clinical routine data without any guidelines, one ward (I) and three outpatient clinics (II, III and IV) participated in the study for five (I, III and IV) or nine (II) random days. They used a new CT and ST each day in parallel. In a few cases, they used only one tourniquet per day. In a questionnaire, the staff documented number of patients, number of personnel worked with the tourniquets, number of disinfection events, disinfection method and unexpected events or peculiarities.

### Tourniquets

The CBC tourniquet (Kimetec GmbH, Ditzingen, Germany) made of polyester and Lycra® (CT) and the ST *daisygrip* (daisygrip GbmH, Rostock, Germany) were used. New tourniquets were used for each day.

### Disinfectant wipes

For reprocessing, tourniquets were wiped with microzid® universal wipes premium (Schülke, Norderstedt, Germany) or Schülke wipes soaked with terralin® protect (Schülke, Norderstedt, Germany; used by facilities II, III and IV).

### Sampling and microbiological investigations

At the end of the working day, tourniquets were sampled at the inner (skin contact) side of the tourniquet with approximately 2 cm distance to the buckle using blood agar contact plates (diameter 5.5 cm, BioMerieux, Nürtingen, Germany). With this method, an area of approximately 13 cm^2^ of the tourniquets was covered. Plates were incubated at 35 °C for 48 h and number of bacteria was determined (indicated as colony forming units (cfu)). After, tourniquets were reprocessed according to the wipe manufacturer’s instruction and sampled again (area next to the first sampled area).

### Statistics

The non-parametric Kruskal-Wallis Test was pairwise performed using the online tool https://www.socscistatistics.com/tests/kruskal/default.aspx; the significant level for null hypothesis testing was set to α = 5%, *P* Values < 0.05 were marked with asterisks.

## Results

In the first trial, we instructed the personnel about the reprocessing procedure to get an impression of the contamination level on conventional (CT) and silicon tourniquets (ST) in routine clinical practice after 1 day of use. In setting (1), staff was asked not to process tourniquets at all, which probably reflects the real scenario as it has been shown that tourniquets were rarely disinfected [3, 6, 16, 17]. In setting (2), the personnel was instructed to treat tourniquets after each use with disinfectant wipes. It should be noted, that this method is not recommended by the manufacturer of CT. But based on our assessment, it is the most suitable method for quickly reprocessing a tourniquet between patients. Each tourniquet has been used between four and 16 times (Suppl. Figure S2).

**Fig. 1:**
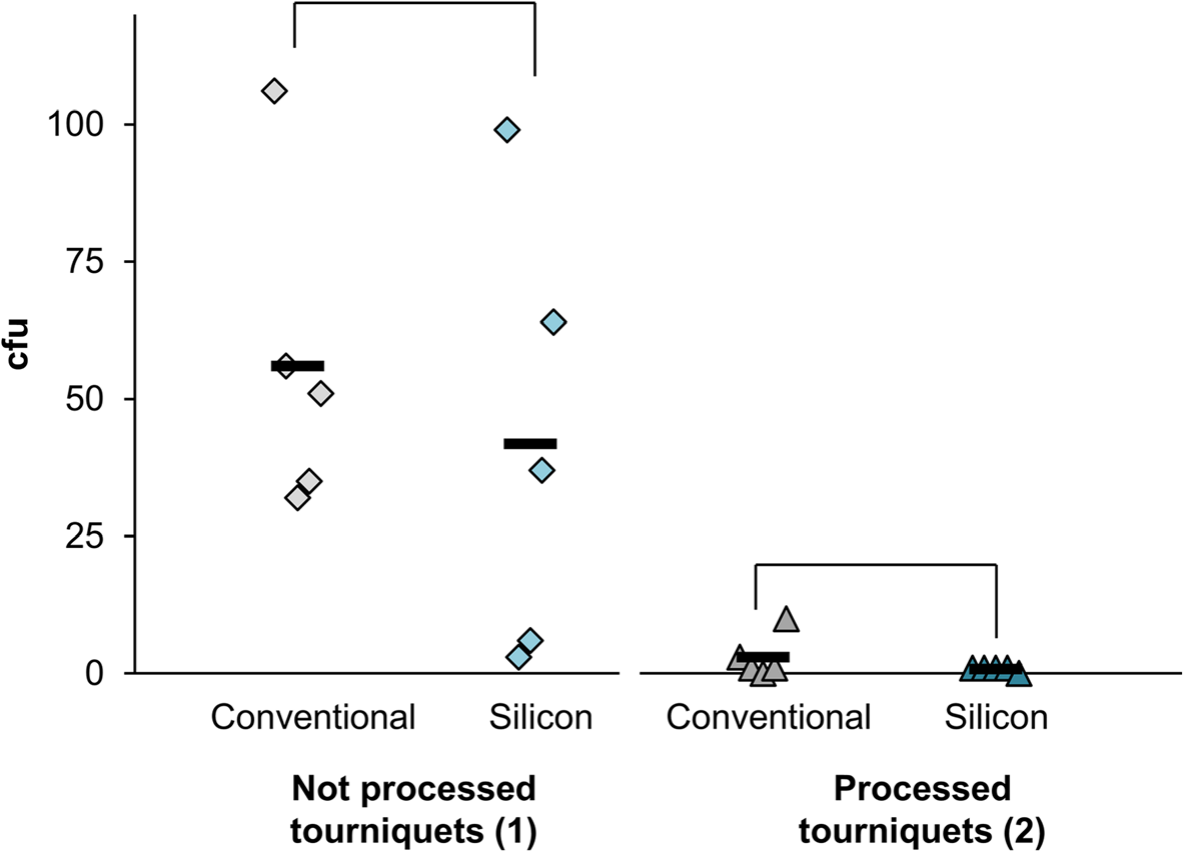
First trial: Bacterial load on different (treated) tourniquets. In one outpatient clinic, new (conventional: grey, silicone: petrol blue) tourniquets were used for 1 day and subsequently sampled at the inner side of the tourniquets. They were either never cleaned (diamond, light) or cleaned after each use with disinfectant wipes (triangle, dark). Individual values (symbols) with averages (black bar) were indicated as colony forming units (cfu) per contact plate. Pairwise Kruskal-Wallis Tests within each setting are demonstrated with brackets

The microbiological assessment of not processed tourniquets (1) showed with certain degree of variation in the contamination level slightly less bacteria (~ 25% reduction, not significant) on silicone tourniquets (Fig. 1). Interestingly, in setting (2) only a very small number of bacteria were detected (< 5 cfu) (Fig. 1) on both tourniquet types, indicating that frequent wiping was not only effective in reducing bacteria on ST but also on CT. In contrast, using disinfectant wipes on not processed tourniquets from setting (1) after sampling was completed, showed distinct more bacteria on CT than on ST (fourfold higher, not significant; Suppl. Figure S3).

The second trial included four facilities and should reflect the procedure in routine practice more than in the first trial. In routine, there are no strict guidelines for reprocessing as in the first trial. Further, there is no recommendation for cleaning conventional tourniquets with disinfectant wipes. In addition, the fabric turned wet after wiping – a condition that could be perceived as uncomfortable for the patient when re-used immediately. Thus, we provided tourniquets together with the manufacturers’ instructions only to avoid biasing the staff towards one particular method of reprocessing.

**Fig. 2:**
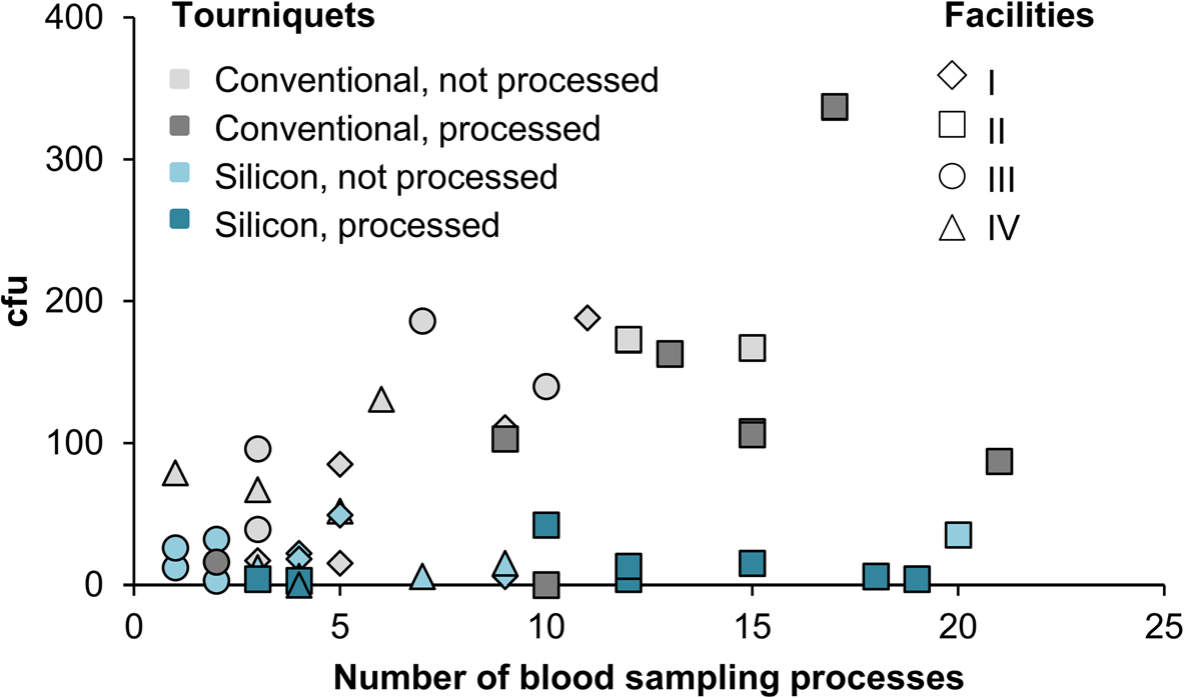
Blood sampling events and tourniquet contamination. Tourniquets were used for 1 day and bacterial load determined on the inner sides. For each tourniquet, the number of venous blood sampling processes was documented and plotted against the respective colony forming units (cfu) per contact plate. The facilities were indicated with symbols (I: diamond, II: square, III: triangle, IV: circle), tourniquets with colours (conventional: grey, silicone: petrol blue) and when tourniquets were cleaned once per day, they were highlighted in darker shade

In all four facilities, the staff processed tourniquets only once per day or not at all. Considering that WHO recommends reprocessing between each patient [18], results showed that the processing frequency of tourniquets is not optimal and confirms findings from earlier studies [3, 6, 16]. The questionnaire indicated similar handling patterns for both tourniquet types, which were always reprocessed using disinfectant wipes although it is not recommended by the manufacturer of the CT. The number of patients varied between one and 21 per day (Fig. 2).

**Fig. 3:**
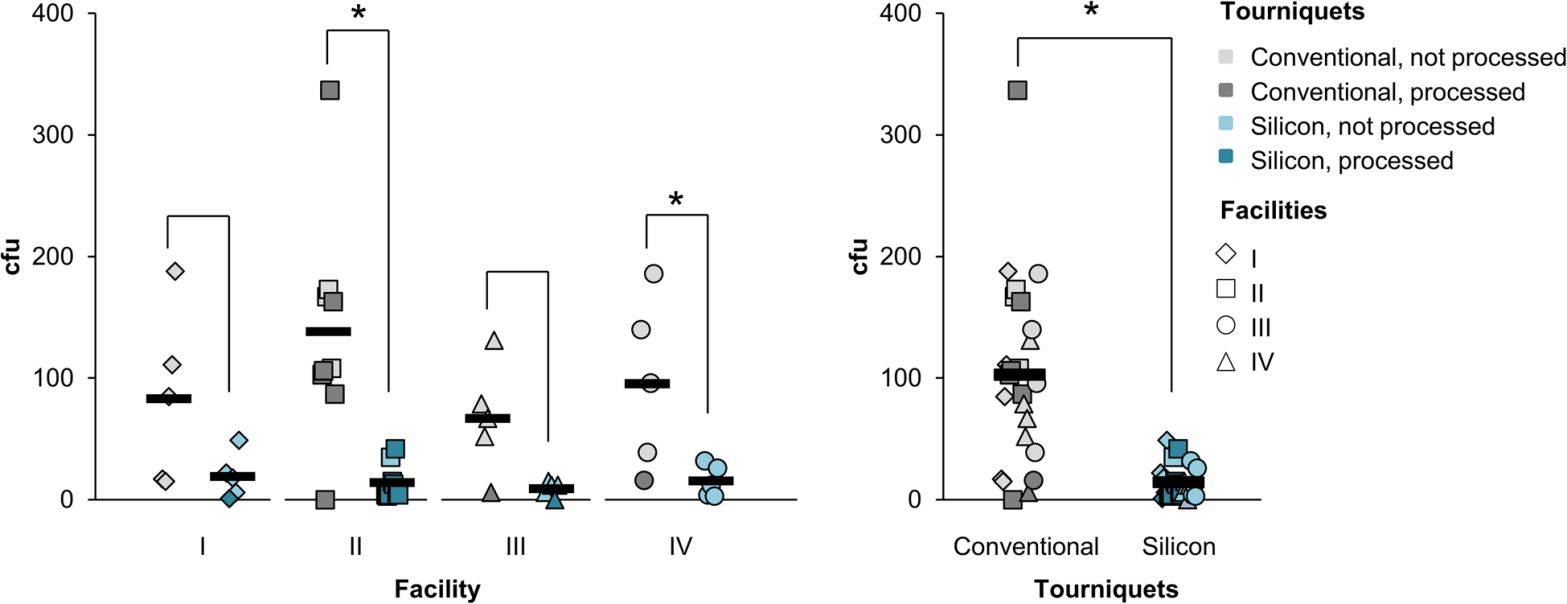
Contamination level on conventional and silicone tourniquets. After 1 day of usage, tourniquets were collected and bacterial load on the inner side of the tourniquet determined. Values are indicated as colony forming units (cfu) per contact plate from conventional (grey) and silicone (petrol blue) tourniquets. Individual values from each facility I-IV (left) and in total (right) are plotted together with their averages (black bar). Tourniquets that were cleaned during the day were highlighted in darker shade. Pairwise Kruskal-Wallis Tests within each facility and in total are demonstrated with brackets and *P* values < 0.05 were marked with asterisks

Despite all tourniquets were similarly processed, contamination levels differed distinctly between the different types of tourniquets in each facility and in total (Fig. 3, Suppl. Figure S4). With a noticeable variance, bacterial loads on CT were significantly higher than on ST (between 77 and 90% reduction in each facility, significant in II and IV; in total 86% reduction, significant). The number of blood sampling processes seemed to play a subordinate role in this context (Fig. 2).

**Fig. 4:**
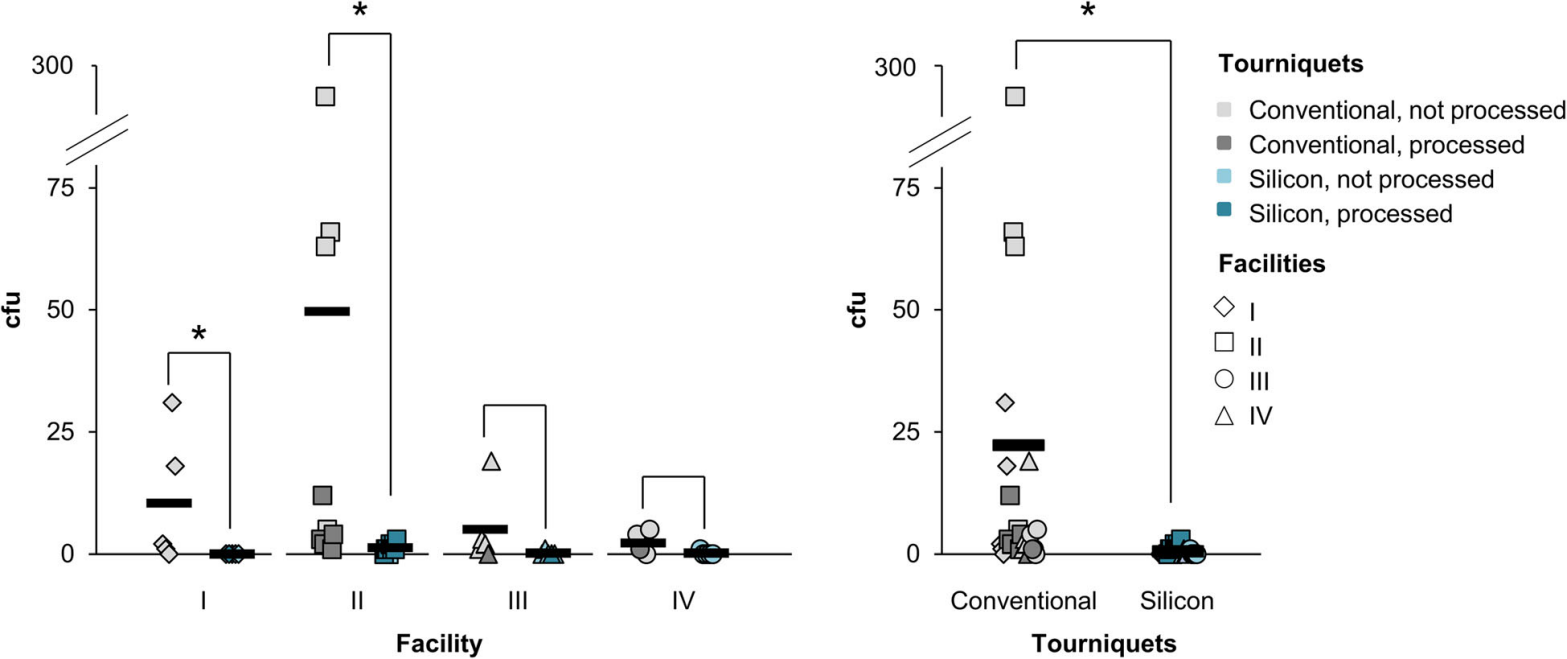
Cleaning tourniquets with disinfectant wipes. After sampling, tourniquets were wiped using disinfectant wipes and subsequently after evaporation sampled again with contact plates next to the original sampling area. Individual values of conventional tourniquets (grey) and silicone tourniquets (petrol blue) with their averages (black bars) were indicated in colony forming units (cfu) per contact plate. Tourniquets that were cleaned during the day are highlighted in darker shade. Pairwise Kruskal-Wallis Tests within each facility and in total are demonstrated with brackets and *P* values < 0.05 were marked with asterisks

Further, data showed that once-daily reprocessing is not sufficient to persistently decrease the bacterial load, as contamination levels between processed and unprocessed tourniquets were similar (Figs. 2 and 3). Reprocessing using disinfectant wipes reduced bacterial load on both tourniquets, but as expected, it was significantly more effective on ST. Data from reprocessed CT showed several samples with particularly striking higher contamination than on ST (Fig. 4).

In summary, ST were less contaminated than CT in both trials. The observed effect was much stronger in the less controlled second trial. Further, ST were not reprocessed more often than CT if no exact instructions were provided although the manufacturer of the ST recommends wiping disinfection after each patient to avoid transmission of infectious agents.

## Discussion

Previous studies have demonstrated the presence of potentially harmful bacterial pathogens on conventional tourniquets (CT) [19], which is why the use of disposable tourniquets was recommended by several authors [2, 3, 6]. However, using disposable tourniquets is neither sustainable nor economical. This study thus investigated whether reusable silicone tourniquets (ST) are indeed superior over CT in terms of infection control considerations. As previous studies demonstrated the frequent presence of potentially pathogenic bacteria [1, 3, 7–13, 17] on these medical products, this study focused on quantitative bacterial contamination as surrogate indicator of risk. We confirmed that wiping ST with disinfectants, which is faster and easier as autoclaving or incubation in disinfectants, is effective in reducing the bacterial load and therefore the potential risk of contracting an infectious agent. Despite being a non-critical medical device (contact only with intact skin) the WHO guidelines for blood sampling recommend reprocessing tourniquets between patients [18]. However, this is not applied in clinical routine [3, 6, 16]. Interestingly, all participating facilities in this study used disinfectant wipes also for CT reprocessing, even though this process is not recommended by the manufacturer. Indeed, this preferred usage has previously been reported [5]. The fast and easy application, the possibility of subsequent usage within minutes and, that disinfecting with disinfectant wipes is well-established in clinical practice, are likely reasons for this approach. The first trial showed that frequent (after each use) wiping of CT is rather effective, while residual contaminations remained frequently on those by cleaning only once per day. Results of the second trial confirmed this finding and revealed that wiping disinfection is more effective on ST. We reason that this easy-to-disinfect property of silicone is based on its hydrophobic smooth surface, which is apparently easier to clean than the rough-surfaced materials of traditional elastic tourniquets.

In the second trial, staff reprocessed both tourniquets with the same frequency, even though fast processing is easier with the silicone due to the material as suggested by the manufacturer’s instructions. These results could indicate that a specific instruction for disinfection is necessary in order to ensure compliance with steady processing. Otherwise, it is possible, that all staff wanted to treat both tourniquets equally because they knew that they were part of a study. To verify these potential explanations, one should monitor the reprocessing on a random basis in institutions where those tourniquets are regularly used.

Data showed that the ST carry less bacteria than the CT after 1 day of usage, though they were not more frequently processed. The effect was more pronounced in the second trial than in the first trial with strict recommendations of reprocessing. This might suggest that this effect is stronger in ‘real’ practice. As there were also different manifestations of the effect between the facilities of the second trial, it could be possible that the deviation of the preliminary trial is caused only by variation between different facilities.

There was no obvious correlation between the number of blood sampling processes and the contamination level being in accordance with the study of Schulz-Stübner et al. where no correlation between the bacterial load and duration or frequency of use was noticed [5]. The number of blood sampling processes of this present study was similar to the range found in other studies [19].

Altogether, ST harbour minor bacterial contamination being 1 day field-tested and can thus contribute to a higher infection control standard. Improved infection control measures of tourniquets should not only improve infection control of blood sampling but moreover any peripheral venous access especially the process of placing peripheral intravenous catheters where the catheter is left in place for several days. One recent study described indeed a correlation between disposable tourniquets combined with other dressings and peripheral intravenous catheter-related contaminations [20], but further studies are needed to define the specific impact of tourniquet contamination in this context. While our study does not investigate this effect, it clearly provides information regarding contamination level and processing of different types of tourniquets. Although one limitation in this regard might have been the short duration of our study as each tourniquet was only used for 1 day. Further studies in clinical practice focusing on the long-term application of different tourniquet types are therefore necessary. However, it is complex to show and understand the link between tourniquet contamination and infection rates. This is outside the limits of an application study with limited control over other factors such as hand hygiene, which was also reported being poor in the process of blood sampling [1, 7, 17, 21, 22] and play a role for infection prevention. Therefore, hygienically optimized tourniquets can only be a piece of the puzzle of the entire process of infection control in peripheral venous vascular access.

## Conclusions

As the bacterial load on silicone tourniquets is in clinical routine distinctly less than on not or rarely reprocessed conventional tourniquets, usage of such tourniquets offers a safer, sustainable and economical alternative to conventional tourniquets in order to improve the infection control standard in the process of blood sampling and other venous vascular access.

## Supplementary information

**Additional file 1: Figure S1.** Conventional and silicone tourniquets. **Figure S2.** Blood sampling events and tourniquet contamination in the first, controlled trial. **Figure S3.** Cleaning tourniquets with disinfectant wipes in the first, controlled study. **Figure S4.** Exemplary blood agar contact plate from tourniquets.

## Data Availability

All data generated or analysed during this study are included in this published article (and its supplementary information files).

## Abbreviations

CFU: Colony forming unit
CT: Conventional tourniquets
HAI: Hospital acquired infections
MRSA: Methicillin-resistant *Staphylococcus aureus*
ST: Silicon tourniquets
