# Supplementary material for: Reduced bacterial contamination rates detected on silicone tourniquets compared to conventional tourniquets in clinical routine
Source: BMC Infect Dis. 2020 Mar 26;20:247. doi: 10.1186/s12879-020-04975-y (PMC7098146; doi:10.1186/s12879-020-04975-y)
Supplement: Supplementary file 1 — Additional file 1: Figure S1. Conventional and silicone tourniquets. Figure S2. Blood sampling events and tourniquet contamination in the first, controlled trial. Figure S3. Cleaning tourniquets with disinfectant wipes in the first, controlled study. Figure S4. Exemplary blood agar contact plate from tourniquets. [file 12879_2020_4975_MOESM1_ESM.pdf]

Supplement

Reduced bacterial contamination rates detected on silicone tourniquets compared to conventional tourniquets in clinical routine

Marcus Grohmann, Lena Schomakers, Frank Wolschendorf, Janina Grosch, Susan Lindner and Anna Kristina Witte

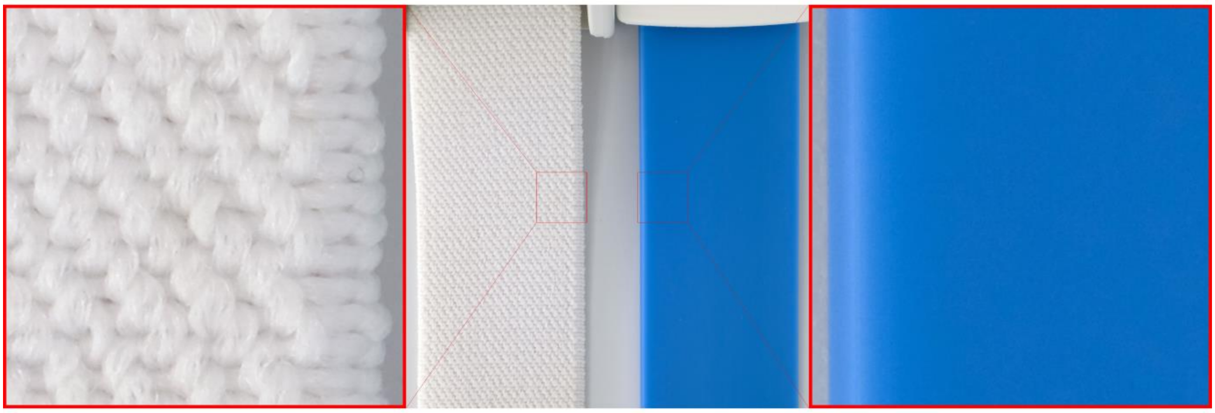

Supplemental Figure S1. Conventional and silicone tourniquets. In contrast to the conventional tourniquets (left, white), the silicone tourniquet (right, blue) consists of an easy to clean hydrophobic and smooth surface.

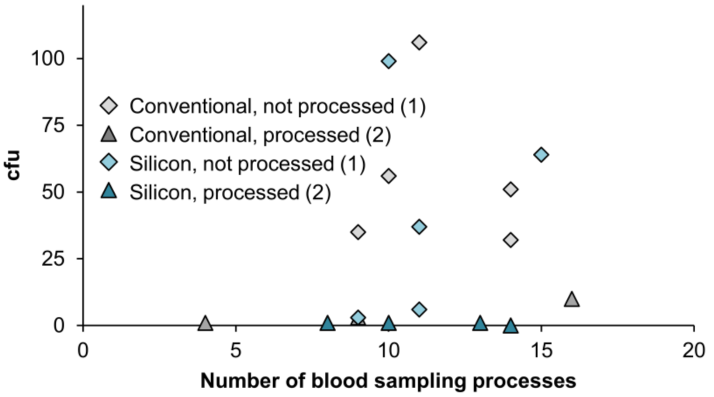

Supplemental Figure S2. Blood sampling events and tourniquet contamination in the first, controlled trial. Tourniquets were used for one day and bacterial load determined on the inner sides. For each tourniquet, the number of venous blood sampling processes was documented and plotted against the respective colony forming units (cfu) per contact plate. Tourniquets (conventional: grey, silicone: petrol blue) were either not cleaned (diamonds, lighter shade) or cleaned after each use with disinfectant wipes (triangle, darker shade).

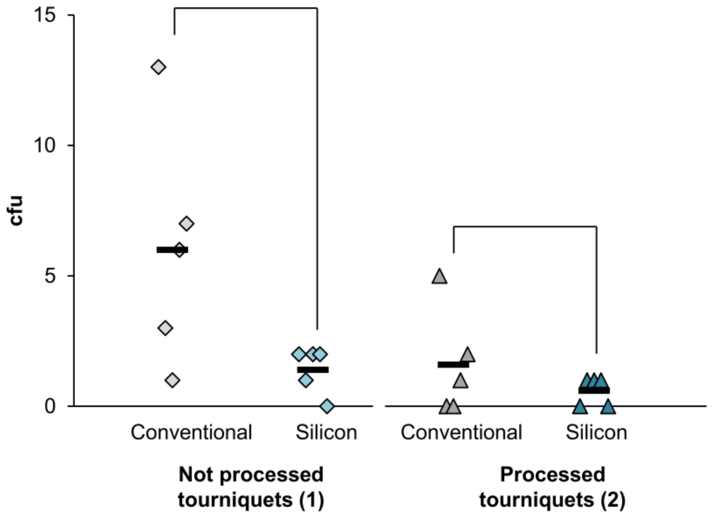

Supplemental Figure S3. Cleaning tourniquets with disinfectant wipes in the first, controlled study. After sampling, tourniquets were wiped using disinfectant wipes and subsequently after evaporation sampled again with contact plates next to the original sampling area. Individual values of conventional tourniquets (grey) and silicone tourniquets (petrol blue) either not cleaned (diamonds, lighter shade) or after each blood sampling process (triangle, darker shade) with their averages (black bars) were indicated in colony forming units (cfu) per contact plate. Pairwise Kruskal-Wallis Tests within each setting are demonstrated with brackets.

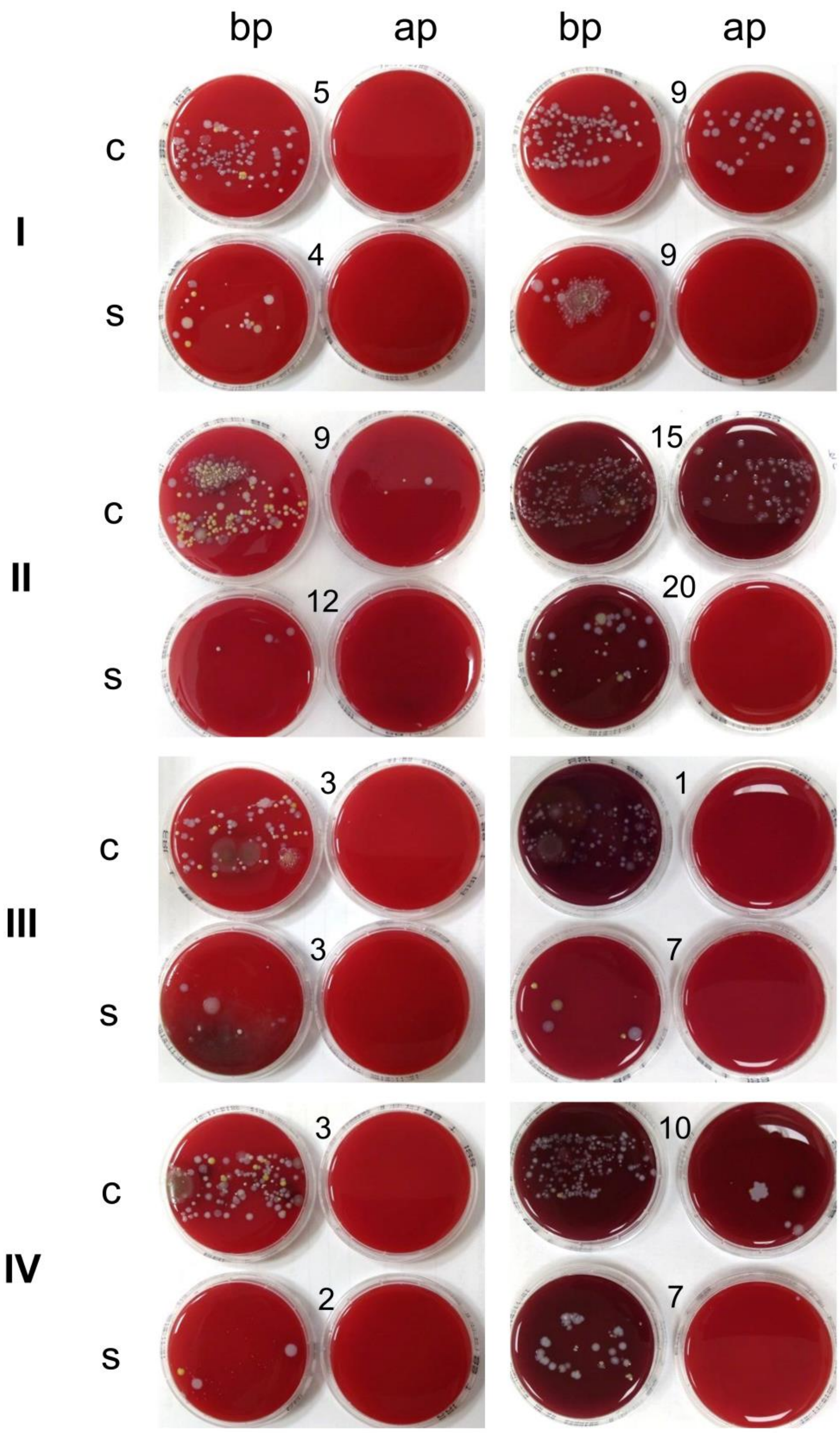

Supplemental Figure S4. Exemplary blood agar contact plate from tourniquets. Contact plates from two days with two sampled tourniquets (c: conventional, s: silicone) of each facility (I – IV) are shown. Contact plates were used directly after collecting the tourniquets (bp: before processing) and after processing them with disinfectant wipes (ap: after processing). The number of blood sampling events is indicated on each set.
